# In search of existential health in Swedish primary care: perspectives from older adults who took part in an existential group treatment

**DOI:** 10.1080/02813432.2026.2696275

**Published:** 2026-07-08

**Authors:** Isak Erling, Carl Anton Waltersson, Margda Waern, Maria Tillfors, Sara Hed, Maria Åberg, Stefan Wiktorsson, Anne Ingeborg Berg

**Affiliations:** a Department of Psychology, University of Gothenburg, Gothenburg, Sweden;; b Slottsskogen Primary Care Centre, Närhälsan, Region Västra Götaland, Gothenburg, Sweden; c Department of Psychiatry, Institute of Neuroscience and Physiology, University of Gothenburg, Gothenburg, Sweden; d Department of Psychotic Disorders, Sahlgrenska University Hospital, Region Västra Götaland, Gothenburg, Sweden; e Department of Social and Psychological Studies, Karlstad University, Karlstad, Sweden; f Department of Neuropsychiatry, Sahlgrenska University Hospital, Region Västra Götaland, Gothenburg, Sweden; g General Practice/Family Medicine, School of Public Health and Community Medicine, Sahlgrenska Academy, University of Gothenburg, Gothenburg, Sweden

**Keywords:** Existential health, existential therapy, existential group treatment, primary care, older adults, psychological distress, qualitative analysis

## Abstract

**Background:**

Although interest in existential perspectives on health is growing, little is known about how such perspectives are experienced by older adults in primary care. The aim of this study was to understand how older adults who took part in a primary care-based existential group treatment describe the role of existential challenges in relation to their health and in primary care.

**Methods:**

Seventeen patients (75+) with psychological distress who had completed a 7-week existential group treatment took part in individual interviews. Transcripts were analyzed using thematic analysis.

**Results:**

Three main themes were derived from the participants’ experiences: (1) Existential challenges are embedded in health in later life; (2) Existential challenges are not usually addressed in primary care consultations; and (3) Engagement with existential challenges is possible within primary care. Older adults with psychological distress describe existential challenges in terms of age-related losses which raise questions of health-related agency. However, barriers to addressing existential challenges in this care setting persist in the form of time constraints, perceived ageism and a one-sided focus on physical health. Relational and trustful healthcare encounters helped to overcome these barriers, as did the existential group treatment.

**Conclusion:**

While existential challenges are embedded in older adults’ health, addressing these issues is not yet an integrated part of primary care services. In search of existential health in Swedish primary care, relational and trustful healthcare encounters as well as structured existential interventions, were found to facilitate the integration of existential perspectives in older adults’ primary care experience.

## Introduction

The term ‘existential’ has become more common in Scandinavian healthcare journals over the past decades [1] and several qualitative studies have focused on healthcare professionals’ experiences of addressing existential perspectives with patients in primary care [2–4]. Although the term ‘existential’ has been applied in somewhat different ways across various contexts, a recent attempt to provide a broad definition has emerged based on an overview of its use in academic health literature [1]. According to this definition, the term ‘existential’ in the context of health entails ‘the basic conditions of being human, the dynamics of suffering and reorientation, meaning and meaninglessness and, finally, secular, spiritual and religious worldviews’ [1]. The increasing interest in existential perspectives on health is further reflected in the Swedish government’s 2024 decision stating that Sweden’s public health work needs to take existential health more seriously [5], and in its assignment to the Swedish Public Health Agency to develop the work on existential health within the framework of the national public health policy [6]. As the first-line healthcare setting for both physical and mental conditions, primary care would make a highly relevant starting point for initiatives to incorporate existential health into regular services.

Previous studies have focused on healthcare professionals’ experiences of addressing existential perspectives with patients in primary care in relation to universal life conditions [2], meaning in life [3], and in questions about what patients find important in relation to being ill [4]. Existential perspectives have been analyzed in relation to general practitioners (GPs) [2] as well as other health care professionals [3], concluding that GPs seem to integrate existential perspectives in their work, but in mostly intuitive and non-standardized ways, and that existential conversations often remain a challenge for health care professionals. The existential therapist Irvin Yalom [7], who is highly cited in Scandinavian healthcare journals [1], developed an existential psychotherapeutic framework in which the basic conditions of being human are understood to give rise to inevitable challenges in life, which can be conceptualized as ‘existential challenges’. Existential challenges have been argued to be especially prominent in old age, in relation to coping with the approach of death and parting, as well as dealing with illness and suffering [8]. Existential challenges such as coping with loneliness, death anxiety and developing a sense of meaning in life, have been linked to subjective wellbeing and mental health in general [9–11] and have been considered to be of particular importance in the context of older adults [12–14]. Research also suggests that some older adults express a need and desire to engage in existential conversations with health care professionals about their mental health [15]. While older adults have a higher consumption of primary care than younger adults [16] access to psychological treatment for older adults with mental health issues is still scarce in the primary care setting [17]. Previous studies among various patient populations including hospital inpatients [18], oncology patients and nursing home residents [19], indicate that existential perspectives can be important to address in health care. At the same time, these studies highlight that the willingness and ways to engage in existential challenges with healthcare professionals vary, and further research is needed to understand the role of existential challenges for patients in the primary care setting.

This study is part of a larger project, *Talking about aging in primary care*, a randomized controlled trial (RCT) which tests a 7-week primary care-based existential group treatment for older adults with psychological distress [20]. Older adults in the current study were interviewed after having completed the treatment and could thus describe their experiences of both regular primary care and the structured research intervention. The research question for this study is: How do older primary care patients, with psychological distress who have taken part in an existential group treatment, describe the role of existential challenges in relation to their health and in the context of primary care?

## Method

This is a qualitative study based on interviews with participants from the ongoing RCT-project *Talking about aging in primary care* [20]. All participants in this study had completed existential group treatment during the first round of the RCT in 2023. They were recruited from five participating primary care centers located in a major city in western Sweden and some surrounding rural communities. An analysis of these participants’ experiences of change processes during the group treatment is presented in a previous publication [21].

### Participants

The first 18 participants who completed the 7-week existential group treatment were asked if they would like to participate in an individual interview and all but one agreed to take part. At the time of recruitment to the RCT all participants were aged 75 years or older and had some degree of psychological distress. The central inclusion criterion in the RCT was self-reported psychological distress at the initial screening, defined as scoring 3 or more on the General Health Questionnaire (GHQ-12) [22]. The Mini-International Neuropsychiatric Interview (M.I.N.I.) [23] was used to classify ongoing mental disorders and to identify exclusion criteria: mania, psychosis, post-traumatic stress disorder, and severe alcohol use disorder. Exclusion criteria also included a clinical diagnosis of dementia or a score of 25 or lower on the Mini-Mental State Examination (MMSE) [24]. Physical comorbidity was self-assessed using the organ-system categories of the Cumulative Illness Rating Scale-Geriatrics (CIRS-G) [25]. For a detailed description of participant characteristics, see Table 1.

**Table 1.** Participant characteristics (*n* = 17).

**Gender**		
	Woman	14
	Man	3
**Age**		
	Median (range)	79 (75–84)
**Living arrangement**		
	Living alone	13
	Living with someone	4
**Marital status**		
	Married	8
	Divorced	6
	Widowed	3
**Place of Birth**		
	Sweden	15
	Nordic countries	0
	Non-nordic countries	2
**Education level**		
	14 years or more	11
	10–13 years	5
	9 years or less	1
**Ongoing physical problems severely affecting everyday life** ^a^		
	Joints, muscles and skeleton	9
	Lower stomach	4
	Eyes, ears, nose and throat	4
	Cancer	4
**Psychiatric conditions** ^b^		
	Major depressive disorder	2
	Panic disorder	1
	Alcohol use disorder	1
	Past episode of major depressive disorder	12
**Suicide risk** ^b^		
	Moderate	1
	Low	4
	No risk	12
**Indications for prescribed psychopharmacological treatment**		
	Sleeping problems (zolpidem, zopiclone and mirtazapine)	8
	Pain (mostly paracetamol)	8
	Anxiety (mostly oxazepam)	5
	Depression (escitalopram, citalopram and venlafaxine)	3
	Alcohol-related problems (naltrexone)	1
	No pharmacological treatment	4
**Global cognitive function** ^c^		
	Mean (SD):	28.7 (1.12)

*Note*. ^a^Self-assessed using the organ-system categories of the Cumulative Illness Rating Scale-Geriatrics; ^b^According to the Mini-International Neuropsychiatric Interview; ^c^According to the Mini-Mental State Examination.

### The existential group treatment

The existential group treatment is a primary care-based intervention for older adults with psychological distress, developed by Erling and Waltersson. The treatment was developed in 2017 with inspiration from Irvin Yalom’s existential psychotherapy [7] and focuses on supporting older adults in handling existential challenges in the process of ageing. Age-related themes such as story of life, freedom, loneliness and death are addressed in the treatment as part of the therapeutic process. A more detailed description of the existential group treatment and participants’ experiences of change processes during this therapy form can be found in a previous publication [21].

The existential group treatment was offered in seven weekly group sessions with 4–6 participants in each group and conducted by two trained therapists employed at each participating primary care center. Group sessions lasted 90–120 min. Before recruitment, all participants received information about the existential group treatment where its aim was described as providing an opportunity to discuss important themes related to ageing and to develop new ways of handling age-related challenges.

### Procedure

Three to five months after fully completing the group treatment, participants were personally invited by letter and phone to be interviewed about the role of existential challenges in primary care and their experiences of the treatment. All participants received verbal and written information about the study from the two first authors. Participants were informed that their participation was voluntary and that they could withdraw at any time without providing a reason. Interviews were conducted by a psychology student who followed a semi-structured interview guide. Prior to data collection, the psychology student was trained in qualitative interviewing including role-play exercises based on the interview guide, by a senior researcher with experience in qualitative interviews with older adults as well as psychologists experienced in working with older adults. The first author listened to the recording of the first interview and provided initial feedback. The interviewer was supervised by the first two authors throughout the interview phase. Informed consent was obtained from all participants and no compensation was provided. Participants chose where the interview would take place, either in their homes or at their primary care center. Interviews were audio-recorded and professionally transcribed. Ethical approval was obtained from the Swedish Ethical Review Authority (2022-02971-01 and 2023-03104-02). Participants were informed that they could contact either of the first two authors by the study telephone if any discomfort should arise during or after the interviews. However, such a need did not arise.

### Semi-structured interviews

The semi-structured interviews included questions on the participants’ experiences of challenges related to ageing, whether they had been able to talk about existential challenges in primary care, and how they think Swedish primary care could better support older adults (see Interview Guide in Supplementary Material). Open-ended questions were employed with follow-ups in order to deepen and further explore participant’s responses. When presenting questions regarding existential challenges, the interviewer sometimes described the concept by referring to themes related to the group treatment (freedom, loneliness and death). Other than that, no formal definition of the term was employed. To help visualize questions about participants’ experiences of physical, psychological and existential challenges in relation to their health and primary care, flashcards representing these three concepts were used during one part of the interview. Sufficient time and a coffee break were provided to ensure that participation was a positive and manageable experience for the participants. Total duration was approximately 1.5 h.

### Analyses

Transcripts were analyzed with thematic analysis in accordance with Braun and Clarke [26]. All interviews had previously been read by the first two authors for an earlier published study [21], but for the present study all interviews were re-read with explicit attention to this study’s research question. The first two authors independently coded all data to avoid the risk of overlooking relevant data and thereafter agreed on a preliminary thematic structure. Following this coding procedure, all co-authors met to review the preliminary themes and thematic structure, and collaboratively worked to refine the themes. This collaborative process ensured that the final themes were firmly grounded in the original codes and directly addressed the research question, capturing the participants’ experiences of the role of existential challenges in relation to their own health and in the context of primary care. In addition, the interviewer read the final analysis to ensure that the findings were consistent with her experience of the interviews.

### Reflexivity

In thematic analysis, researchers’ own positions and assumptions are recognized as inevitably influencing the interpretation of data [26]. With the two first authors being developers of the existential group treatment and practitioners in primary care, theoretical perspectives on existential therapy as well as clinical experiences might affect the analysis. Throughout the analytical process, these positions were reflected upon and discussed between the authors. This study also drew on the diverse expertise of all co-authors, including clinical psychology, general practice, gerontology and geriatric psychiatry, to provide a broad understanding of the data. The approach to existential therapy developed by Yalom and its view of life as consisting of basic existential concerns [7] influenced the development of the existential group treatment. Although this was not an explicit theoretical starting point, it may have indirectly informed the analysis.

## Results

As shown in Table 2, the analysis yielded three main themes: (1) Existential challenges are embedded in health in later life; (2) Existential challenges are not usually addressed in primary care consultations; and (3) Engagement with existential challenges is possible within primary care. Six related subthemes were also generated. Each main theme is presented below, followed by subthemes with quotes that illustrate the participants’ experiences.

**Table 2.** Summary of main themes and subthemes.

Main themes	Subthemes
1. Existential challenges are embedded in health in later life	
	1.1 Age-related losses bringing awareness of life’s finitude 1.2 Questions of agency
2. Existential challenges are not usually addressed in primary care consultations	
	2.1 External barriers of time pressure, ageism and one-sided physical focus 2.2 Internal barriers to help-seeking and individual preferences
3. Engagement with existential challenges is possible within primary care	
	3.1 In relational and trustful healthcare encounters 3.2 Through a structured existential format

### Existential challenges are embedded in health in later life

The first main theme describes the participants’ experiences of existential challenges in everyday life and how these challenges were embedded in their general health situation. Existential challenges were not described as separate phenomena but rather as integrated parts of one’s well-being. Participants’ existential challenges emphasized questions about how to manage one’s health condition rather than distinct reflections on general life concerns such as identity, life fulfilment or meaning in life. The first subtheme emphasizes how existential challenges are related to health among older adults with psychological distress through *Age-related losses bringing awareness of life’s finitude*. Losses in the participants’ lives as older adults evoked reflections on their health situation along with an awareness that their remaining time is limited. The second subtheme emphasizes how existential challenges are embedded in health in terms of *Questions of agency,* raising reflections on possibilities and limitations related to ones’ general health condition.

#### Age-related losses bringing awareness of life’s finitude

Age-related losses such as losing loved ones, adapting to deteriorating bodily functions, as well as experiencing loneliness were present in most participants’ descriptions of their process of seeking help at the primary care centers for psychological distress. These losses were often related to experiences of death or approaching death in the face of life’s finitude. The deaths of loved ones and weakening of the own body affected the participants’ general health situation. One participant described her own experience of getting older and how age-related losses affected her mental health:

This inevitable thing is that good friends become ill. My sister and my brother and sister-in-law and so on, they’ve all died… and then it’s the friends who get all sorts of ailments… There’s a lot of talk about this, or you check up on each other and things like that. And then you’re reminded of your own frail body. And also, very much that you don’t want to lose them… There is some sort of background anxiety here… ‘What will it be like in five years?’… It is a challenge in many ways to get older.

Relational losses involved experiences of both caring for and losing a partner, and many also told of how losing friends affected them. In this sense, losses were described as part of everyday life for many participants. One described how he became increasingly cognizant of ageing and death:

And now we come to the existential thing that is going to drag on. It becomes very tangible. Now you read obituaries and you check, when did they die? It’s not interesting who it was, but when did they die, or rather how old were they when they died, when were they born? They are in the 1930s so far and then one or two in the 1940s. Damn, now I’ll see if I’ll survive all these obituaries.

Seemingly irreversible age-related ailments, such as memory loss, a weakening body and diminishing amount of energy were further described as affecting the participants’ health.

There are limitations when you’re old, you get that automatically with old age. Well, you don’t have the same speed anymore. You don’t have that energy like getting up quickly and thinking ‘Now I’m gonna do it’. That doesn’t really exist anymore. When you wake up in the morning you have to think ‘Today I’m going to do this and today I’m going to do that’. Then you have to see what you have the strength for… if it’s enough for anything else?

For some participants, loss of loved ones and loss of own bodily functions were understood as somewhat similar processes. Both evoked conscious reflections on how to handle losses in relation to an awareness of life′s finitude. As one participant put it:

You lose people, but you also lose parts of yourself. There’s a lot to say goodbye to.

#### Questions of agency

When speaking of existential challenges, many participants described how age-related losses raised thoughts related to agency in relation to their own general health condition. These thoughts on agency were manifested as becoming aware of what is important in life, consciously prioritizing those values, and assuming responsibility for the time one has left. As one participant put it:

In which end should one start? These physical problems, you try, you get hearing aids and you take medications. But this thing about losing words and such, you try to solve crosswords and Sudoku and whatever it can be to keep it all together, but when you don’t get restored, you try to take long walks, you try to do things, but even that becomes limited. So, the physical condition makes the whole situation revolve around psychological aspects. And then you start to think, damn, how old am I really? And now I’m not talking about actual age but really, what can I do and what can’t I do anything about?

Some participants described further how their physical and mental health problems raised questions regarding what is important in life and whether life with age-related losses is worth living. One participant who had survived several episodes of life-threatening illness described how these episodes evoked existential challenges regarding agency and priorities in life:

It becomes existential. These were life-threatening illnesses. And then there is always this, if I have the privilege of having x number of healthier years… you think, what do I want? What do I want to get out of these years?… And that is a challenge.

For another participant, self-reflections on agency evolved around the issue of whether life was worth living:

Well, it’s these physical problems that have come in these last years. Atrial fibrillation, vision loss, problems walking, problems with balance, and things like that… With these physical problems you end up with the existential problem of like, is it worth it?

For some participants the term ‘existential’ was not a natural word in their everyday vocabulary. Nevertheless, they tended to use the concept as reflecting on their general health condition, rather than as separate life issues. Deteriorating bodily functions and awareness of own mortality evoked thoughts related to the question *How can I act on this*? In this sense age-related losses were described as existential challenges in the participants’ lives when these problems started self-reflective processes, stressing the role of own agency related to health.

### Existential challenges are not usually addressed in primary care consultations

The second main theme describes the participants’ experiences of seeking help in primary care and the barriers of addressing existential challenges in this context. Barriers were mainly described as structural hinders within the health care system, but some participants also brought up internal barriers. Two subthemes emphasize how existential conversations are hindered in primary care due to *External barriers of time pressure, ageism and one-sided physical focus*, as well as *Internal barriers to help-seeking and individual preferences*.

#### External barriers of time pressure, ageism and one-sided physical focus

Many participants described experiences of primary care as not fully being able to attend to their needs as older adults with psychological distress. Existential challenges generally went unnoticed by health care professionals due to structural barriers within primary care and many also described a lack of focus on psychological aspects of health in general. One participant described her first experience of expressing a need to talk to someone in primary care:

If we’re going to discuss what it’s like being in primary care, I’ve been going to a physiotherapist for years. I then tried to talk with my doctor about this and that I was not feeling so well and the only thing she had to suggest was, well you can go to our primary care rehab. And then I felt like how I just sank… Then someone knocked on the door just as I was about to start saying that I needed to talk to someone… So, I just burst out crying and then when [the doctor] comes back she doesn’t take hold of it, despite seeing that I was sad, she said ‘So here’s the phone number for rehab’. And I just said, ‘Thank you’, thanks for nothing basically, and left. So, I was really disappointed with my doctor, that she didn’t even [react]. She saw, she ought to have seen. But then I didn’t have the strength to deal with it. I have a pretty rough tongue so I basically thought, [expletive] you, that’s it.

Many participants described the short duration of primary care consultations as a major barrier to existential conversations. Awareness of the time pressure had a negative impact.

The problem with the primary care center is that they don’t have time. You experience, and if you’re like me, very verbose on some occasions, you see how their faces get more and more wrinkled when the allotted half hour has passed. So, one gets concentrated on the physical problems. That’s what it’s about… Talking about existential questions? That’s not possible.

Perceived ageism was another phenomenon that came up when discussing barriers to communication from a broader health perspective that went beyond a focus on physical health. Some participants experienced that primary care services are not adapted to meet the needs of older adults. Others described experiences in which they felt that their health problems were neglected due to their age.

If a younger person walks around unhappy, it seems unnatural and we call for the doctor and the psychologist. If an older person walks around sad, it’s more the course of nature… I think this is very ingrained. It’s a belief that we have. Most of us know that this is not fun getting old, of course not. So, the healthcare system lets go of older people.

Many participants mentioned that a narrow perspective on health that focused on physical conditions left little room for attention to psychological challenges in general and existential challenges in particular. Existential challenges were generally perceived as not being part of regular primary care.

I don’t think you can turn to the primary care centre about this. I don’t think so. I haven’t received any information about this… You visit the primary care centre when you are sick, physically sick, that is.

#### Internal barriers to help-seeking and individual preferences

In relation to structural barriers within primary care, participants also described experiences of self-restraint and individual preferences that could inhibit existential conversations within primary care. One participant described how he wanted more focus on the existential questions related to his health condition but how he himself tended to say that all is well and focus on his physical ailments during primary care consultations:

I think just raising these questions could do a little bit… These existential questions… When I see the doctor ‘How are you?’ And what do I or all of us answer in ninety-nine cases out of a hundred: ‘Good. But I have a pain in my toe’. You immediately move on to the physical aspect… When these ten or fifteen minutes have passed, the doctor would ask ‘Was there anything in particular you were here for?’ ‘Yes, I had a pain in my toe but right now it doesn’t feel that bad′… Then they should handle these aspects with you as an old person. Everything from ‘What have you done today? Could you have done anything differently?’ And all that, to get these existential parts to work. That’s what you could wish for from primary care. But I have a feeling that they have a whole heap of bad toes and how much is your cholesterol today?

One aspect of not addressing existential challenges seemed linked to the dominant physical focus within primary care, that affected some participants’ own ability to seek help from a broader health perspective. Some participants described processes of internally restraining one’s own needs by not addressing anything else than physical health concerns in primary care. As one participant put it:

If I felt deeply depressed, I wouldn’t turn to primary care… Then I would start looking for a private psychologist or something else. It wouldn’t cross my mind that primary care would be relevant in this context… They deal with bodies at the primary care centres.

The ability to engage in existential challenges in primary care was also affected by some participants’ own attitudes of not wanting to take too much time as they were cognizant of the limited resources in primary care.

I don’t contact primary care to get counselling about something that I feel they don’t have time for. It just doesn’t exist. There are people who have it much worse and [the primary care centres] doesn’t have the resources. So, it doesn’t come naturally, no.

Internal barriers to help-seeking was also manifested in more general attitudes of how to cope with life’s problems. Some participants possessed personal conventions of keeping a stiff upper lip and holding things for oneself, rather than engaging with difficult issues with healthcare professionals. One participant described how she had taken care of herself from an early age, never sharing her problems with others. This long-term approach to life’s struggles affected her ability to bring up existential challenges during primary care consultations in later life:

I’m used to keeping everything to myself… [Talking about existential challenges in primary care] hasn’t existed in my world. It was like when you worked back then; you had so much, imagine if you could ask for help? But I didn’t. I gritted my teeth… this has been going on since childhood.

Several participants expressed that they hadn’t recognized a need to engage in existential challenges in primary care before taking part in existential group treatment. Some participants described experiences of not directly thinking about existential challenges in life, whereas others already felt able to handle existential challenges by themselves. For participants who were used to holding things for themselves, a certain relational connection or trust would be needed if they were to share their existential challenges. The term “existential” was also a bit unclear for some participants. As one participant put it:

Well as for existential challenges. I don’t really know if I have experienced that word, existential. I think that existence that it’s me who is the existence, if you put it that way, and that I’ve had challenges. But if it’s been existential? I’m not sure about that. But I’ve had my share of challenges over the years.

### Engagement with existential challenges is possible within primary care

The third main theme describes the participants’ experiences of engaging with existential challenges in primary care and how they appreciated talking about their health from a broader health perspective. Factors that facilitated engagement with existential challenges included individual health care professionals’ own abilities to reach over barriers of time, ageism and a one-sided focus on physical health, providing a more relational encounter, as described in the first subtheme *In relational and trustful healthcare encounters.* Engagement with existential challenges were also facilitated by the structured approach they had experienced in the form of existential group treatment. This was described in the second subtheme *Through a structured existential format.*

#### In relational and trustful healthcare encounters

Some participants described experiences in which they had felt listened to by individual healthcare professionals from a broader health perspective that went beyond their physical health status, and how this had a positive impact. One participant described her positive experience with a general practitioner (GP) who made room for her existential challenges in relation to her physical condition and thereby provided a broader perspective than she was used to in primary care:

Well, the doctor I have now whom I like very much, he told me the last time we talked to each other, that we have to keep an eye on you so that you don’t fall into a depression. So, he was keeping a bit of a lookout. But I think he’s unusual. I have never experienced a doctor asking questions about my painting and what it means to me and so on. It’s the first time… He took a whole hour and talked with me, so he is a very good example.

In relational and trustful health care encounters like these, some participants described important experiences of being able to speak about their health condition from a broader health perspective which affected them positively. In this sense, enabling engagement with existential challenges within regular primary care depended in many cases on specific relations with health care professionals. With certain people, engagement with existential challenges was expressed as possible, whereas with others, participants described experiences of neglect. Participants who had positive experiences of engaging with existential challenges with a particular healthcare professional also described how shifting from a trusting relationship with that person to another professional had been negative for them.

There was a Greek doctor there and well, it was a joy to be sick with him. Unfortunately, he is no longer there, and since then I haven’t gone back. It would have been very good because you could actually talk with him. He saw the whole person. He was very interested in your physical ailments as well as your own thoughts and so on. And unfortunately, not everyone is.

When articulating the needs of older adults in primary care, many participants emphasized the impact that certain individual relations with specific doctors, nurses and therapists had for their experiences of primary care. While all such relationships were considered important, there was a notable emphasis on how relationships with physicians in particular shaped participants’ experiences of primary care. Consultations characterized by continuity and familiarity were described as fundamentally different from meeting a new physician each time. One participant who had struggled with a string of different GP’s described her take on how primary care can become better at supporting older adults with psychological distress:

Listen, listen, listen. And have something to say. And not just say, ‘Take paracetamol’… You have to see the whole person. An old person needs care just like a little baby. The more you give a small child, the more you get back. The more you give an older patient, the more that person can cope without having to move into a care home and cost an awful lot of money.

Engaging in existential conversations within primary care was in this sense not described as something that happened by itself. Rather, it required a relational and trusting connection with a healthcare professional. As one participant put it:

These conversations don’t arise by themselves. It takes a sensitive person to initiate them, to frame them, and to decide that this should be talked about. These are rare topics of conversation, no matter how much it weighs on each of us in our solitude.

#### Through a structured existential format

When describing their experiences of participating in the structured existential group treatment, many brought up feelings of being recognized in a new and different way. All but one of the participants expressed that there is a need for such a format in regular primary care. One participant stressed that it provided a way to get relevant help without having to think of herself as mentally ill.

I think it’s a good treatment… it’s a huge difference for most people to seek psychiatric care compared to getting something like this at their primary care centre… Here I’m offered something to help me deal with a period in my life. I don’t have to be sick, I don’t have to think of myself as sick. But I’m facing a task to take care of myself in a new phase.

Several participants expressed that they appreciated the broader perspective on older adults’ health situation. One participant described her reaction when she first heard about the opportunity to engage in a research study involving existential conversations in primary care:

Well, how interesting, have they started to take an interest in us in this way?… Not just like patching up the body. So, I thought right away, of course I’ll join this.

In relation to engaging in existential challenges in a structured format within primary care, several participants described how existential conversations are quite rare due to fears and possible tabus related to these topics in other contexts. One emphasized how a structured and directed format focusing on existential challenges in primary care enabled conversations that wouldn′t typically emerge in other primary care consultations:

Things like this you don’t talk about. That’s probably one of the great benefits of this group treatment and the idea of starting it. In what other contexts do you start talking about death? If you join a club, you don’t say ‘Hello, I feel like doing away with myself’. No, here you need a guiding hand and someone who makes it permissible to bring up these difficult topics. And a doctor can certainly ask some cautious questions, ‘How are you doing?’ and so on, but nobody really takes that bait.

Several participants shared reflections on how a structured existential group format could make a difference for older adults with psychological distress in primary care. Some suggested it to be beneficial in reducing loneliness issues, and some further suggested that a broader health perspective might reduce care costs. One participant described her reason for seeing a need for this format for older adults within primary care in the following way:

If they join a group [like the existential group treatment], I think that might improve their mental health. Because everyone benefits when people are taken care of, all people of course, but now we’re talking about older adults. If one becomes more mentally calm and harmonious, then one can handle ageing. You don’t get as many ailments, and if you get an ailment you try to deal with it and I think that’s really important. It saves time, it might save home care, medical care, the whole lot. Everyone benefits from this.

Structured existential group treatment was in this sense described as possible within primary care and filling needs for older adults with psychological distress that were not easily met in regular primary care. In this context, one participant described how structured existential group treatment might make a difference for patients if it could become a part of primary care as natural as physical healthcare:

I think this would make a difference… A group treatment can be just as important as physiotherapy… It would be great if referring someone to a discussion group about life problems became as natural as referring someone for a bad back. My way of initially holding back, is completely related to the prejudices I have. Emotions aren’t something you talk about and that’s not the doctor’s area, and thank you very much, I’ll handle that myself. I mean, if one can manage to somehow dedramatize this. Because these are very big words we’re talking about: Existence, freedom and death.

## Discussion

This study shows how older adults with psychological distress find existential challenges embedded in their general health situation but that existential perspectives are not yet an integrated part of regular primary care. Older adults with psychological distress describe existential challenges in terms of age-related losses which raise questions of health-related agency. Barriers to addressing these issues in primary care involve time constraints, a one-sided focus on physical health, and perceived ageism whereby participants expressed feeling that their health problems were at risk of being neglected due to their age. Participants also addressed internal barriers that reduces the likelihood of disclosure of mental health issues. General practitioner (GP) consultations that include an existential approach are characterized by relational presence as well as a more holistic approach to health, both of which were described as positive among participants. The structured existential group intervention was appreciated as a means to dedramatize mental health and to increase own agency in managing age-related issues in later life. Together these findings suggest that existential health initiatives can be integrated into primary care in order to better address older adults’ late life situation.

From a lifespan perspective, existential challenges were present in everyday life for many of the participants through reflections on own agency related to age-related losses. These reflections were manifested in a process of becoming aware of what is important in life, prioritizing those values, and assuming responsibility for the time one has left. Existential challenges expressed among participants in this study align with developmental psychology theories such as Baltes and Baltes’ lifespan model of Selection with Optimization and Compensation (SOC) [27], in which a deliberate utilization of SOC-strategies is proposed to be fundamental for an effective adjustment to health-related and psychosocial losses in late life. In previous literature, existential health has been understood by Sigurdson [28] as a reflective experience related to health in general and Binder [29] has further highlighted that suffering is an unavoidable aspect of life, which suggests that actively relating to suffering needs to be regarded as a fundamental aspect of health. This understanding of existential health aligns well with the narratives of the participants in our study, stressing the need for primary health care professionals to understand the concept of existential health in a manner that makes room for patients’ own health-related reflections in later life. In relation to the overall definition of the term ‘existential’ in the context of health proposed by Nygaard et al. [1], it is interesting to note that the participants’ experiences resemble this definition in relation to “the basic conditions of being human,” “the dynamics of suffering and reorientation” and “meaning and meaninglessness,” but to a lesser extent in relation to “secular, spiritual and religious worldviews.”

The participants in this study expressed little room for engagement with existential challenges in regular primary care due to several barriers. Previous studies have demonstrated that older adults are often underrepresented in utilization of mental health services in general [30,31] and that ageist attitudes, both societal and internalized, risk contributing to inadequate care for older adults [32,33]. In a Swedish primary care study, persons aged 20–66 with mental health problems also described insecurity and uncertainty in this care context, and a feeling of disinterest on the part of the GP was described by some [34]. Older adults in our study seemed to implicitly adapt to external barriers and tended not to ask for help for their mental distress and existential challenges, which increased the negative effects of time pressure, ageist attitudes, and the one-sided focus on physical health within primary care. Continuity and adequate time allocation for patients and health care professionals alike might therefore be central prerequisites for improving primary care consultations for older adults in general and successful existential health initiatives in particular.

This study also highlights examples of primary care encounters that enabled room for older adults’ existential challenges in contrast to routine care. Primary care consultations with an existential approach were described as relational, trustful and engaging, and seem to align with the principles of person-centered care (PCC) [35]. With the aim of improving patient adherence and treatment outcomes, PCC emphasizes individual choice, autonomy, and respect for each patient’s own values and preferences and has been considered to be an important approach for improving primary care [35] and well-being for older adults [36]. From a person-centered and holistic perspective, older adults’ existential reflections may thus be viewed as a resource in the healthcare encounters. Just as healthcare providers cannot regard PCC as an optional add-on to ordinary services, existential health may also need to be understood as a broadened perspective on primary health care in general. Finally, all but one of the participants in this interview study agreed when asked whether a structured group treatment like the one they experienced during the research intervention should be made available for older adults in routine primary care. Several participants emphasized how the structured and directed format enabled conversations about existential challenges that would not otherwise happen in the primary care setting. The existential group treatment was described as helping to dedramatize mental health and support older adults in adapting to health-related challenges, thereby illustrating potential benefits of existential health initiatives in primary care. A forthcoming publication will expand this line of research by testing whether the structured existential group treatment may also lead to measurable health-related benefits such as increased quality of life and decreased psychological distress [20].

## Limitations

All participants in this study were recruited from an active arm in an ongoing RCT, *Talking about aging in primary care* [15]. Before being interviewed all participants had completed existential group treatment, which enabled them to describe experiences of engaging in existential challenges within a structured format as well as within regular primary care. Although this approach made the participants purposely and carefully selected for the research question, the results need to be understood in this context. All participants had chosen to be part of the RCT, indicating that they were open to participate in existential group treatment, but because of the randomization procedure, they could not actively choose the existential group treatment. The fact that randomization occurred after study inclusion thus reduced the likelihood that participation in the intervention was solely based on a specific preference for existential group treatment. However, participants’ responses were presumably colored by their experiences during the existential group treatment, and older adults who have not taken part in such an intervention might be less equipped to verbalize existential challenges in relation to their health and in the context of health care. Further, both care availability and care processes for older adults with symptoms of psychological distress may differ widely in various primary care treatment settings in a global context, presumably also affecting perceptions of the role of existential challenges in health and primary care. Since the sample in this study was relatively homogeneous, with a predominance of women born in Sweden, the findings cannot be directly extrapolated to older adults with more diverse demographic characteristics.

## Clinical implications

In order to successfully implement existential health initiatives for older adults in primary care, healthcare providers need to make more room for patients own health-related reflections in routine care. Relational and trustful health-care encounters, along with specific existential interventions, appear to facilitate existential conversations in primary care and support older adults in adapting to health-related challenges in later life. To better attend to patients’ existential challenges, particular emphasis should be placed on avoiding fragmented and overly time-limited healthcare encounters. Educational initiatives might also support GPs in integrating existential communication more naturally into everyday clinical practice [2].

## Conclusion

Older adults with psychological distress have in this study described existential challenges as embedded in their general health situation but attending to these existential challenges are not yet a natural part of routine primary care. In search of existential health in Swedish primary care, both structured existential interventions as well as relational and trustful healthcare encounters have been found to counteract current barriers and improve primary care for older adults with psychological distress.

## Supplementary Material

Supplemental Material

## Data Availability

The data presented in this article are not readily available because the transcripts cannot be sufficiently de-identified to ensure participant anonymity, making it unsuitable for sharing. Requests to access the data should be directed to isak.erling@gu.se.

## Clinical trial number

The authors declare that financial support was received for the research, authorship, and publication of this article. The study was financed by grants from the Swedish Research Council, Grant number 2021–06466, the Swedish Research Council for Health, Working Life and Welfare, Grant number 2016–07097, the Swedish government under the ALF Agreement, ALFGBG 1005419, ALFGBG 942661, ALFGBG 1005368 and R&D Gothenburg and Southern Bohuslän, RHS 2024-01272, RHS 2025-01458.
