# Supplementary material for: In search of existential health in Swedish primary care: perspectives from older adults who took part in an existential group treatment
Source: Scand J Prim Health Care. 2026 Jul 8;44(1):2696275. doi: 10.1080/02813432.2026.2696275 (PMC13353439; doi:10.1080/02813432.2026.2696275)
Supplement: Supplemental Material [file IPRI_A_2696275_SM7808.pdf]

Supplementary material: Interview guide

Initially, I will ask you some questions about your life situation and your experiences of primary care *before* you joined the existential group treatment.

1. For about half a year ago you chose to take part in the study “Talking about aging in primary care”. What was it that made you want to participate?
    - How would you describe your life situation when you said yes to participate in this?
    - Which challenges related to aging did you experience when you said yes?
2. Have you tried before to talk about challenges related to aging at primary care centers. If so, in which way and how was the experience?

Here are some challenges that one may experience related to aging [shows and explains three cards with the topics: Physical issues, Psychological issues and Existential challenges]:

- Which of these challenges have you experienced related to aging, and which do you feel you have been able to talk about at the primary care center?
- Do you feel that these challenges are related in any way? If so, how?
- Have you turned to other settings to talk about existential challenges related to aging? If so, which ones?"

Now I am going to ask you a few questions about the existential group treatment that you have participated in.

1. If you think back just before you started in the existential group treatment. The first time when you were about to go to the first meeting, what went through your head?
    - Do you remember if you had any expectations about what you were going to take part in? If so, what expectations did you have?
    - Did you think that it would be helpful for you to participate in this? If so, in which way and why?
2. How did it then turn out for you to participate in the existential group treatment?
3. When you participated in the existential group treatment, which challenge or difficulty in aging was important for you to talk about?

4. Has your approach to this particular [challenge/difficulty] changed in any way after your participation in the existential group treatment?

    - Can you give any example from your life in how you have handled this [challenge/difficulty] differently after the existential group treatment?

5. Are there any other areas in your life where you have experienced any type of changes after the existential group treatment?

    - Can you give any example from your life in how you have handled [other areas] differently after the existential group treatment?

6. Has your attitude towards challenges/difficulties in life changed in *general* after your participation in the existential group treatment?

7. If you now think back on the existential group treatment that you have participated in. Would you like to share a situation that was particularly significant for you during the existential group treatment?

Break with coffee

Now we will talk a bit more about eventual changes one might experience when participating in an existential group treatment like this, and which aspects of the treatment you think can contribute to such changes.

1. Do you feel that the existential group treatment contributed to any changes in your *well-being*, and if so, how? Change can be both positive or negative.

2. Insofar that you have experienced any form of change in your well-being, what do you think it was *in the existential group treatment* that contributed to this?

    - Was there any other component in the existential group treatment that you think affected your well-being?

3. Now we will try to explore this question a bit further with the help of some cards. Different aspects of an existential group treatment can have varying importance for different people, and here I have five different components that may influence one's well-being in existential group treatment [Shows five cards with the topics: Own engagement and expectations, The group situation, The therapists, The working material, and The existential themes]. I would now like to discuss with you which of these were important for eventual changes you experienced. There are no right or wrong answers.

Supplementary material: Interview guide

After we've now talked about your experiences from the existential group treatment, I would like to ask you three final questions.

1. Do you see a need for this kind of initiative within primary care. If so, in what way and why?

2. How do you think Swedish primary care could improve in supporting older adults with challenges related to aging?

3. Finally: Is there anything that we have not talked about that you would like to share?

Thank you so much for your participation!
